# Is KingVision videolaryngoscope with a bougie really an effective solution for emergency intubation?

**DOI:** 10.1186/s13054-018-2259-7

**Published:** 2018-12-05

**Authors:** Fu-Shan Xue, Rui-Juan Guoa, Liu-Jia-Zi Shao

**Affiliations:** Department of Anesthesiology, Beijing Friendship Hospital, Capital Medical University, NO. 95 Yong-An Road, Xi-Cheng District, Beijing, 100050 People’s Republic of China

The recent article of See et al. [1] introducing the bougie-in-channel intubation technique shows that the use of a KingVision videolaryngoscope with a bougie can result in high first-attempt success in manikin and human trials. However, their manikin and human studies were small single-arm observable studies without a control design. Thus, an important question that remains unanswered in this study is whether this new technique really performs better than other methods for emergency intubation. Other than first-attempt success, moreover, the time required for intubation is also a special concern for critically ill patients requiring emergency intubation, especially patients at risk of hypoxia and aspiration [2]. In their manikin trial, the use of a 2-min cut-off time for successful intubation is evidently inappropriate.

Most importantly, we have several concerns regarding this technique. (a) It may be a time-consuming process and has a long learning curve because a 16-step process is required. (b) Because of a small diameter, the bougie tends to cut across the curve of the guiding channel and pass posteriorly during passage towards the larynx, leading to failure [3]. (c) The available evidence indicates that a mismatch between the mouth opening and the device because of its bulky design is another main reason for failure of emergency intubation using the KingVision device [4]. After the bougie is shifted laterally out of the guiding channel, moreover, the anterior part of the bulky blade close to the larynx can impede advancement of the endotracheal tube along the bougie into the glottis. (d) The KingVision videolaryngoscope has an angulated channeled blade without an option for direct laryngoscopy. For emergency intubation, a videolaryngoscope combining the benefits of direct and video laryngoscopy in one device is often recommended as the most appropriate choice as intubators can immediately switch to another option to successfully complete the intubation without having to make a second attempt after the first option fails [5]. Thus, we believe that large randomized controlled trials are still needed to validate the true role of the bougie-in-channel intubation technique in emergency intubation.
