# Supplementary material for: Is KingVision videolaryngoscope with a bougie really an effective solution for emergency intubation?
Source: Crit Care. 2018 Dec 5;22:334. doi: 10.1186/s13054-018-2259-7 (PMC6280334; doi:10.1186/s13054-018-2259-7)
Supplement: Related texts

# Reply: Authors' Response

**Authors:** See Kay Choong, Estaras Melanie, Capistrano Rolando, Wong Sui Hua, Sahagun Juliet, Taculod Juvel

We thank Dr. Xue and colleagues for their interest in our article [1]. We fully support broader validation of the bougie-in-channel intubation technique and agree that the KingVision videolaryngoscope is unsuitable for direct laryngoscopy.

Dr. Xue and colleagues raised several discussion points. Firstly, the bougie-in-channel intubation technique could be learnt after a short 15-min demonstration of 16 discrete steps. In comparison, direct laryngoscopy with a bougie would be more complex, as additional neck positioning would be required. Moreover, limited neck extension [6] or a very anterior larynx [3] would preclude a straight oral–pharyngeal–laryngeal axis.

Secondly, the bougie did not cut across the curve of the guiding channel using our described method. Dr. Xue and colleagues quoted a case report where videolaryngoscopy was done using the Airtraq device (Prodol Meditec, Vizcaya, Spain) with a mounted endotracheal tube, followed by bougie insertion [3]. Unexpectedly, the bougie penetrated the tube’s Murphy eye, leading to intubation failure. The bougie-in-channel intubation technique would avoid this issue, as the bougie would be inserted *before* railroading the endotracheal tube.

Thirdly, mouth-opening required for the KingVision device was not unusually large, nor did its blade impede endotracheal tube advancement. In a recent study, mouth-opening measurements for the channeled KingVision videolaryngoscope and a non-channeled videolaryngoscope (C-MAC D-blade, Karl Storz, Tuttlingen, Germany) were virtually identical [7]. Conversely, in Cavus et al. [4], mouth opening was not measured. Rather, the predominant problem was difficulty in passing the endotracheal tube within the guiding channel, which the bougie-in-channel intubation technique would overcome.
